# Cerebral Pathology

**Published:** 1882-02

**Authors:** 


### CEREBRAL PATHOLOGY.

The latest general pathological propositions relating to the encephalon have been given by Seguin, as follows:

1. Lesions of the basis cerebri, especially if involving the pons varolii and crura, give rise to the following symptoms: Paralysis, anesthesia in the face and limbs, impairment of equilibrium, changes within the eyes; no psychical symptoms.

2. Lesions of the great basal ganglia probably produce no symptoms unless encroached upon by the internal capsule which passes near them.

3. Lesions of the white center of the hemispheres produce no symptoms when they do not involve the parts composing the internal capsule. If the anterior portion of this capsule be injured, we observe paralysis; if its posterior part, anesthesia.

4. Lesions of the cortex cerebri produce, when located anteriorly, psychical symptoms; when located in the median regions, paralysis of an imperfect kind; and when situated posteriorly, no symptoms at all.

5. Lesions of the cerebellum produce no symptoms except by involving adjacent parts containing important motor and sensory tracts, thus giving rise to irregular paralysis, changes in the optic apparatus, symptoms of irritation of the vagus nerve, etc.

6. Lesions of one-half of any part of the encephalon produce motor and sensory symptoms in the side of the body opposite to the lesion. When the lesion is in one-half of the basis cerebri some symptoms are found in the side of the face and head corresponding to the lesion, others in the opposite half of the body.

7. Lesions in the median line cause symptoms to appear in both sides of the body.

8. Any intra-cranial lesion which acts in such a way as to increase the intra-cranial pressure may produce, in addition to other symptoms, the condition known as choked disk or neuro-retinitis.

